# Mechanism of action of non-coding RNAs and traditional Chinese medicine in myocardial fibrosis: Focus on the TGF-β/Smad signaling pathway

**DOI:** 10.3389/fphar.2023.1092148

**Published:** 2023-02-09

**Authors:** Chunjun Li, Xiangxiang Meng, Lina Wang, Xia Dai

**Affiliations:** ^1^ College of Traditional Chinese Medicine, Shandong University of Traditional Chinese Medicine, Jinan, China; ^2^ College of Marxism, Shandong University of Traditional Chinese Medicine, Jinan, China; ^3^ First College of Clinical Medical, Shandong University of Traditional Chinese Medicine, Jinan, China; ^4^ College of Health, Shandong University of Traditional Chinese Medicine, Jinan, China

**Keywords:** non-coding RNA, myocardial fibrosis, Traditional Chinese Medicine, TGF-β/Smad signaling pathway, mechanism

## Abstract

Cardiac fibrosis is a serious public health problem worldwide that is closely linked to progression of many cardiovascular diseases (CVDs) and adversely affects both the disease process and clinical prognosis. Numerous studies have shown that the TGF-β/Smad signaling pathway plays a key role in the progression of cardiac fibrosis. Therefore, targeted inhibition of the TGF-β/Smad signaling pathway may be a therapeutic measure for cardiac fibrosis. Currently, as the investigation on non-coding RNAs (ncRNAs) move forward, a variety of ncRNAs targeting TGF-β and its downstream Smad proteins have attracted high attention. Besides, Traditional Chinese Medicine (TCM) has been widely used in treating the cardiac fibrosis. As more and more molecular mechanisms of natural products, herbal formulas, and proprietary Chinese medicines are revealed, TCM has been proven to act on cardiac fibrosis by modulating multiple targets and signaling pathways, especially the TGF-β/Smad. Therefore, this work summarizes the roles of TGF-β/Smad classical and non-classical signaling pathways in the cardiac fibrosis, and discusses the recent research advances in ncRNAs targeting the TGF-β/Smad signaling pathway and TCM against cardiac fibrosis. It is hoped, in this way, to give new insights into the prevention and treatment of cardiac fibrosis.

## 1 Introduction

Cardiovascular disease (CVD) is one of the leading causes of death worldwide, causing approximately 32% death in 2019. It is estimated that about 23.6 million people will die from CVD by 2030, imposing a serious economic burden on the world (Ahmed et al., 2023). In contrast, cardiac fibrosis is closely related to the pathological development of almost all CVDs and even can lead to heart failure (HF), seriously affect quality of life of the patient (Xue et al., 2022). Development of myocardial fibrosis is closely related to cardiac fibroblasts, which secrete collagen fibers to maintain the normal structure and function of the heart. Effector molecules of the renin-angiotensin-aldosterone system (RASS) system such as Angiotensin II (Ang II) and aldosterone (ALD) can promote the fibroblasts proliferation and enhance the collagen gene expression, thus developing the cardiac fibrosis (Davis and Molkentin, 2014). In addition, cardiac fibroblasts can be transformed into myofibroblasts induced by various stimuli such as cytokines, pressure load, inflammatory mediators, and mechanical tension. Besides, they can secrete more collagen fibers to promote ventricular remodeling (Li et al., 2022a). Furthermore, cardiac fibroblasts maintain the extracellular matrix (ECM) homeostasis, which is essential for ventricular systolic and diastolic function. When the heart is injured, ECM homeostasis will be disrupted and deposited, causing structural disruption of myocardial tissue, increasing the ventricular stiffness and adverse remodeling, and developing the HF with preserved or reduced ejection fraction (Li et al., 2022a). Despite significant advances in myocardial fibrosis in recent decades, myocardial fibrosis cannot be cured with specific drugs and present an increasing morbidity and mortality of chronic heart failure (CHF) (López et al., 2021). Therefore, better understanding the molecular mechanisms of cardiac fibrosis after myocardial infarction (MI) is conducive to providing new therapeutic targets for inhibiting cardiac fibrosis.

The TGF-β/Smad signaling pathway involves in the initiation of fibrotic responses in several tissues, and is particularly associated with the development of fibrosis after MI (Chen et al., 2022). TGF-β1 is a stimulator which can convert cardiac fibroblasts to myofibroblasts. While classical TGF-β pathway signaling is mediated by the binding of TGF-β1 to transforming growth factor beta receptors II (TGFBR2) (Huang et al., 2022), which subsequently phosphorylates and activates the Smad2 and Smad3, thereby promoting myofibroblast proliferation and migration, and leading to cardiac fibrosis (Zhang et al., 2022a). Non-coding RNAs (ncRNAs) can be broadly classified into microRNAs (miRNAs), long-stranded non-coding RNAs (lncRNAs), and circular RNAs (circRNAs) according to their functions (Iu et al., 2022). The ncRNAs can bind to multiple molecular targets, and form regulatory networks in many biological activities, including initiation of specific cellular biological responses, regulation of gene expression, intracellular signaling, and epigenetic genetic modifications (Statello et al., 2021). In recent years, an increasing number of researches have exhibited that ncRNAs are involved in the development and progression of cardiac fibrosis by regulating the TGF-β/smad signaling pathway (Guo et al., 2022). Furthermore, with unique theoretical and rich resource for over 2,000 years, TCM has been proven to delay the progression of cardiac fibrosis by targeting and modulating the TGF-β/Smad signaling pathway. However, there are fewer systematic reviews on the intervention of ncRNAs and TCM in cardiac fibrosis by modulating the TGF-β/Smad signaling pathway. Therefore, this work focuses on the TGF-β/Smad signaling pathway, and summarizes the biological functions of miRNAs, lncRNAs, and circRNAs that regulating the progression of cardiac fibrosis, particularly in regulating the TGF-β/Smad signaling pathway. Besides, this work highlights the potential that TCM can target the TGF-β/Smad signaling pathway and resist the cardiac fibrosis, which may contribute to the clinical treatment.

### 1.1 Overview of the TGF-β/Smad signaling pathway

**FIGURE 1:**
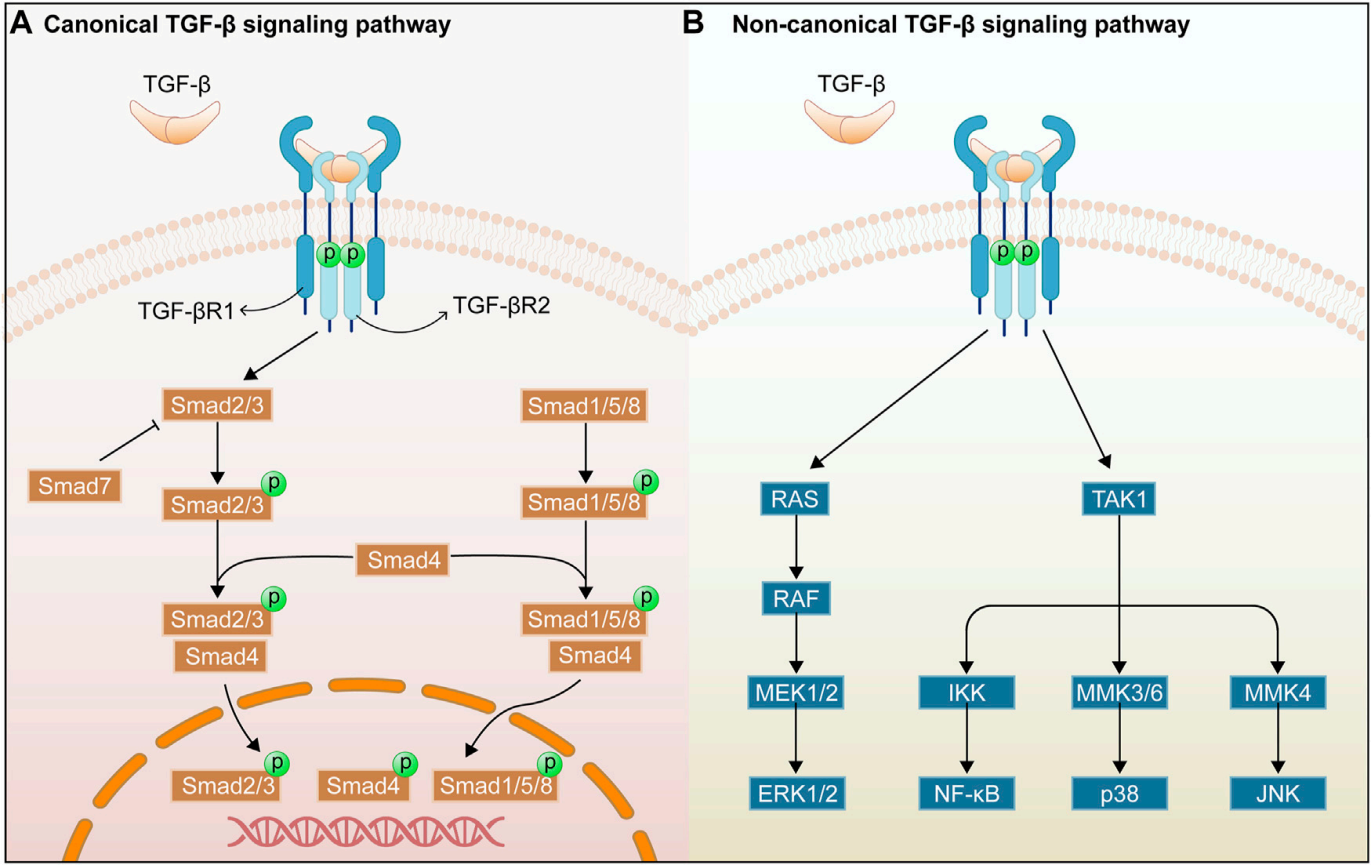
Overview of TGF-β/Smad signaling pathway. Canonical TGF-β signaling pathway **(A)**. Non-canonical TGF-β signaling pathway **(B)**.

There are considerable evidences that the TGF-β/Smad signaling pathway plays a critical role in organ fibrosis, and that the TGF-β receptor and its mediators importantly regulate the organ fibrosis process (Figure 1). TGF-β was first identified as a multifunctional cytokine in the early 1980s, and it is deemed as the transforming growth factors β1 (TGF-β1), β2 (TGF-β2), and β3 (TGF-β3) in mammals. TGF-β1 is the fully-researched member of the transforming growth factor family and is an integral part in tissue fibrosis. This work focuses on the roles of TGF-β1 in the TGF-β/Smad signaling pathway (De Oliveira et al., 2021). TGF-β1 is mainly expressed in endothelial, haematopoietic, and connective tissue cells, where it activates fibroblasts and promotes the synthesis of ECM (Meng et al., 2016). TGF-β2 acts as an indispensable cytokine, similar to TGF-β1, to regulate the cell proliferation, differentiation, migration, and death. Meanwhile, it is a key growth factor in development of fibrogenesis in myofibroblasts (De Oliveira et al., 2021). In addition, TGF-β2 deficiency can affect the epithelial-mesenchymal interactions, cell growth, extracellular matrix production, and tissue remodeling, leading to multi-organ defects (Sanford et al., 1997). TGF-β3 exerts a key role in normal development of the maxilla and lung while involving in epithelial-mesenchymal transition (EMT), cell growth, apoptosis, differentiation, ECM production, and remodeling using an environment-dependent and tissue-specific manner (Kaartinen et al., 1995; Chakrabarti et al., 2020). In addition, TGF-β is secreted as an inactive ligand, which consists of a latency-associated peptide (LAP) and a maturation peptide. LAP is primarily to keep the TGF-β protein in an inactive state and to prevent it from interacting with its receptor. Binding between the TGF-β protein and the LAP can be disrupted by various stimuli such as reactive oxygen species (ROS), integrins, proteases, and metalloproteinases, thereby releasing mature TGF-β protein. Then, the active TGF-β can interact with the receptors of intracellular signaling pathways (Yu et al., 2022).

TGF-β receptors include transforming growth factor-β type I receptor (TGFβRI), transforming growth factor-β type II receptor (TGFβRII), and transforming growth factor-β type III receptor (TGFβRIII). TGF-β1 can bind directly to the TGFβRII receptor, and TGFβRII can recruit and activate the TGFβRI after binding of TGFβRII to TGF-β by phosphorylating the GS structural domain in the TGFβRI (Hachana and Larrivée, 2022). In addition, TGFβRIII increases the bioavailability of TGF-β signaling to TGF-β receptors by transferring TGF-β to its signaling receptor (Yu et al., 2022). In addition, Smad proteins have been extensively studied as key intracellular effectors of TGF-β, playing the role of transcription factors (Meng et al., 2016). The Smad classical signaling pathway is mediated by the Smad protein family of related receptors, which contains eight members in humans (Smads1-8). Besides, it can be divided into three subgroups, namely receptor-activated Smads for Smad2/3, and Smad1/5/8, co-mediator Smad for Smad4, and inhibitory Smads for Smad6 and Smad7 (Luo, 2022). Smad2/3 can be phosphorylated and activated by TGFβRI receptor, and then the phosphorylated Smad2/3 can combine with Smad4, forming a heterotrimer complex and transferring it to the nucleus to regulate the transcription of target genes (Wang et al., 2017). Smad7 is a repressive Smad, and is regulated by the level of phosphorylated Smad3. Moreover, Smad7 competes with Smad3 and Smad2 for the binding site of TGFBR1, thereby promoting TGF-β1/Smad signaling (Mallikarjuna et al., 2022). The Smad non-classical signaling pathways can be triggered by the binding of TGF-β1 and TGFBR2, including p38 mitogen-activated protein kinase (MAPK), c-Jun N-terminal kinase (JNK), extracellular signal-regulated kinase (ERK), and Nuclear factor of κB (NF-κB) signaling pathways (Chen and Chang, 2022). The above non-classical signaling pathways will be discussed in Section 2.

## 2 The role of TGF-β/Smad signaling pathway in cardiac fibrosis

### 2.1 The roles of TGF-β in cardiac fibrosis

TGF-β is one of the drivers of fibrosis in most organs. As reported, members of the TGF-β family played important roles in many processes related to cardiac pathophysiology, including cardiac repair, hypertrophy, fibrotic remodelling, fibroblast activation, and ECM deposition (Lovelock et al., 2005; Bagchi et al., 2016; Shinde et al., 2017). Moreover, TGF-β, a key cytokine in fibrotic events, can stimulate the fibroblast activations, including fibroblast to myofibroblast differentiation, collagen synthesis, and ECM protein deposition (Bagchi et al., 2016). Besides, it can induce the expression of *a*-SMA which is a marker of myofibroblast maturation (Shinde et al., 2017). In addition, TGF-β can inhibit the matrix metalloproteinase activity by producing more matrix metalloproteinase inhibitors, thus reducing the ECM degradation, aggravating deposition, and accelerating the development of fibrosis (Lovelock et al., 2005). Notably, pulmonary arterial hypertension (PAH) increases the endothelial-mesenchymal transition (EndMT) and collagen production, thus promoting the development of cardiac fibrosis. TGF-β family plays a major role in the development and progression of PAH, and is an important regulator in pulmonary vascular remodeling and inflammation, cardiac hypertrophy, and cardiac fibrosis (Ol et al., 2018). Therefore, it is reasonable to assume that TGF-β has an important role in the process of cardiac fibrosis.

TGF-β1, one of the isoforms of TGF-β, is most widely distributed in the mammalian tissues. TGF-β1 plays a vital role in pathological fibrosis and can trigger the development of tissue fibrosis, especially cardiac fibrosis, so it is upregulated in cardiac fibrosis patients and animal models (Nicin et al., 2022). As exhibited in the response to cardiac injury, TGF-β1 can activate the non-fibroblasts, including cardiomyocytes, endothelial cells, and immune cells, thereby conversing the activated fibroblasts and myofibroblasts (Nicin et al., 2022). Moreover, TGF-β1 binds to the TGFβR1 receptor and then activate the Smad2/3 protein, thus leading to collagen production (Lee et al., 2020). Besides, TGF-β1 induces autophagy, and conversion of cardiac fibroblasts to myofibroblasts can be inhibited by inhibiting autophagy (Gupta et al., 2016). In addition, TGFβ2 has been shown to regulate the expression of ECM proteins through the classical and non-classical Smad signaling pathways (McDowell et al., 2013). In contrast, TGF-β3, an important factor in cardiovascular development, mediates the pathological process of CVDs such as cardiomyopathy. In addition, TGF-β3 together with ncRNAs regulate the development of renal cardiac fibrosis in mice (Liu et al., 2022a). Interestingly, not only are the three isoforms of TGF-β involved in the development of cardiac fibrosis, but TGFβR1 and TGFβR2 are integral in the progress of cardiac fibrosis. Conditional deletion of TGFBR1 from fibroblasts significantly attenuates the pressure overload-induced cardiac hypertrophy and enhanced the ventricular function (Khalil et al., 2017). In addition, deletion of TGFBR1 ameliorates the cardiac dysfunction, cardiac hypertrophy, and collagen deposition induced by MI (Chen et al., 2022). The upregulation of TGFBR2 is closely associated with HG-induced cardiac fibrosis, while its downregulation improves the HG-induced proliferation, differentiation, and collagen accumulation of human cardiac fibroblast (HCF) (Li et al., 2016). It has been confirmed that specific ablation of TGFBR2 in myofibroblasts can block the TGFβ signaling in activated fibroblasts, thereby lowering the risks of cardiac fibrosis and cardiac hypertrophy (Meng et al., 2018). Therefore, TGF-β-related factors are closely associated with the cardiac fibrosis, and targeting and regulating the expressions of TGF-β-related factors may be important and effective to treat the cardiac fibrosis.

### 2.2 Roles of Smad2 and Smad3 in cardiac fibrosis

Smad2 and Smad3 are typical transcription factors downstream of the TGF-β signaling pathway, share a similar structure, possess highly homologous amino acid sequences, but exhibit different roles in tissue repair and fibrosis (Meng et al., 2016). Smad3 is a key mediator of TGF-β signaling in ECM production and tissue fibrosis, and Smad3 deficiency in downstream of the TGF-β signaling pathway alleviate the cardiac fibrosis, enhances the cardiac compliance, and insulates diabetic mice from cardiac fibrosis (Dong et al., 2021; Tuleta and Frangogiannis, 2021). Moreover, Smad3 can specifically reduce myocardial collagen content by inducing fibroblasts, thus being conductive to maintaining the ECM synthesis in the heart (Khalil et al., 2017). Meanwhile, Smad3 deficiency may promote the progression of PAH and cardiac fibrosis (Li et al., 2021a). It has also been shown that Smad3-specific deficiency in fibroblasts, rather than Smad2-specific deficiency, can greatly inhibit the cardiac fibrosis in infarcted myocardium (Huang et al., 2019). In contrast, Smad2 is activated in the TGF-β-stimulated fibroblasts and the infarcted myofibroblasts, whereas myofibroblast-specific Smad2 deficiency can temporarily attenuate the post-infarct remodelling (Huang et al., 2019). Fibroblast-specific Smad2 deficiency exhibits no effects on myocardial collagen content, suggesting the little role of Smad2 signaling in activating the fibroblast repair program (Khalil et al., 2017).

### 2.3 Roles of Smad4 in cardiac fibrosis

Smad4, encoded by the Smad4 gene, is an intracellular transcriptional mediator of the TGFβ signaling pathway, so it can interact with Smad2/3 in cells and mediate the TGF-β signaling pathway (Li et al., 2022b). In addition, Smad4, as the only Co-Smad identified in mammals, mediates the signals from TGF-β and bone morphogenetic protein (BMP) signaling pathways and helps them shuttle into the nucleus (Kamato et al., 2013). Activated peroxisome proliferator-activated receptor *?* (PPARγ) retards the development of cardiac fibrosis, which can be inhibited by TGF-β1 by enhancing the binding of Smad4 and histone deacetylase 1 (HDAC1) and weakening the binding of HDAC3 to the PPARγ promoter in cardiac fibroblasts (Gong et al., 2011). Smad4 signaling can induce the apoptosis in cardiomyocytes and specific deletion of Smad4 in cardiomyocytes leads to cardiac hypertrophy, aggravating the myocardial fibrosis (Wang et al., 2005). Similar to the aforementioned contents, Smad4 is lowly expressed in CHF patients and CH model animals, while its upregulation can inhibit the progression of CHF and CH, which in turn can attenuate the progression of myocardial fibrosis (Wang et al., 2022). Thus, Smad4 plays a key role in cardiac fibrosis and inhibited Smad4 may alleviate the cardiac fibrosis by inhibiting the activity of Smad3-responsive promoter.

### 2.4 Roles of Smad7 in cardiac fibrosis

TGF-β1 can exert the biological effects through the activation of Smad2 and Smad3. While the activation is negatively regulated by Smad7, indicating that Smad7 acts as an antagonist in TGF-β signaling and thus exerts an anti-fibrotic effect (Hu et al., 2017). According to the relevant researches, Smad7 expression is downregulated in cardiac fibrosis in diabetic cardiomyopathy (DCM) rats, and the high glucose-induced cardiac fibrosis could be alleviated by promoting the expression (Meng et al., 2019). Similarly, the elevated TGF-β1 expression and reduced Smad7 expression are observed in fibrotic tissues of hypertensive rats, whereas overexpressed Smad7 inhibits the HCFs being differentiated into myofibroblasts, thereby attenuating the cardiac fibrosis (Xiao et al., 2020). Moreover, Smad7 expression is upregulated in MI-induced fibroblasts, and its conversion to myofibroblasts is associated with the dependence on Smad3 signaling. Simultaneously, Smad7 over-expression can weaken myofibroblast transformation and reduce collagen type I (Col-I) and fibronectin synthesis, while not affecting the collagen type III (Col-III) levels (Humeres et al., 2022). Thus, it may be an important potential target to promote Smad7 expression for the treatment of cardiac fibrosis.

### 2.5 The role of other TGF-β-induced non-classical signaling pathways in cardiac fibrosis

TGF-β acts in cardiac fibrosis by activating the TGF-β/Smad classical signaling pathway and by regulating non-classical signaling pathways, such as p38MAPK, JNK, ERK, and NF-κB, thereby affecting the progression of cardiac fibrosis. MAPKs are a large family of kinases associated with important cellular functions, including extracellular signal-regulated kinases (ERK1/2), c-JunN-terminal kinases (JNK1, 2, and 3), and p38 MAPKs (p38α, β, γ, and δ) (Turner and Blythe, 2019). As reported, p38MAPK played a key role in the regulation of fibrosis. Moreover, inhibited p38MAPK can inhibit TGF-β-induced Smad2/3 phosphorylation and *a*-SMA expression, and reduce the pressure overload-induced cardiac hypertrophy and fibrosis in the left ventricle (Turner and Blythe, 2019). Besides, the role of the JNK signaling pathway in fibrosis is confirmed in numerous evidences. For example, interleukin 22 (IL-22) can further increase TGF-β1-induced collagen synthesis *in vitro*, which can be restricted by inhibitors of the JNK pathway. This work implies that IL-22 may promote fibrosis *in vitro* by activating the JNK signaling pathway. TGF-β1 also can increase the phosphorylation levels of ERK1/2 in fibroblasts, and the inhibition of ERK expression blocks collagen gene expression in cardiac fibroblasts (Yang et al., 2015). Thus, by inhibiting the factors involved in the MAPK signaling pathway, the progression of cardiac fibrosis can be delayed. Similar to the researches above, NF-κB activation plays a key role in the progression of cardiac fibrosis, for example, AngII induces the NF-κB activation and promotes the cardiac fibroblasts proliferation and collagen synthesis, while cardiac fibrosis can be alleviated effectively by inhibiting the NF-κB activation and nuclear translocation (Gupta et al., 2016; Zhou et al., 2021).

## 3 Regulation of TGF-β/Smad signaling pathway by ncRNAs in cardiac fibrosis

### 3.1 Overview of ncRNAs

The ncRNAs are a class of genes with limited or without protein-coding capacity, representing 60% of human transcripts. ncRNAs were previously considered to be the “dark matter” of the genome (Kapranov et al., 2010). However, the important regulatory functions of ncRNAs in numerous biological processes, such as cell proliferation and adhesion, apoptosis, angiogenesis, and human migration, were gradually revealed in the last decade (Beermann et al., 2016).

The ncRNAs can be classified into small ncRNAs (sncRNAs, 18-200 nt), lncRNAs (>200 nt), and circRANs with a unique loop structure, based on the 200 nt cut-offs of their mature transcript length (Bian et al., 2021). The small ncRNAs can be divided into small nuclear RNAs (snRNAs), small nucleolar RNAs (snoRNAs), miRNAs, and piRNAs (Pinho et al., 2022). In the following introduction, miRNAs, lncRNAs, and circRNAs are focused. The miRNAs are a broad family of sncRNAs with approximately 18–25 nucleotides in length, which are primarily involved in the gene regulation at the post-transcriptional level and target at the specific mRNAs by binding to the 3′UTR of mRNAs in full or partial complementarity (Gebert and MacRae, 2019). LncRNAs are a group of ncRNAs with more than 200 nucleotides in length and multiple regulatory roles (Mercer et al., 2009), with biogenesis similar to mRNAs. lncRNAs are transcribed by RNA polymerase II, followed by the capping in the 5′region and the polyadenylation in the 3′region (Yang et al., 2022). In addition, lncRNAs are present in almost all organisms, represent a high proportion of RNAs in complex organisms, and are expressed at different levels in different tissues (Zhang and Wang, 2022). The circRNAs are a new type of ncRNAs widely existed in animals and other organisms, and a special closed loop can be formed by the reverse splicing between downstream splice donors and upstream splice acceptors (Guo et al., 2014). As indicated by the researches, circRNAs played multiple roles in different subcellular compartments, such as transcriptional activation, post-transcriptional regulation, translation, and protein interactions (Li et al., 2018). Besides, circRNAs can further participate in protein mediation by regulating transcription *via* the competitive sponge-like miRNAs (Lin et al., 2022).

### 3.2 Regulation of TGF-β/Smad signaling pathway by miRNAs in cardiac fibrosis

Cardiac fibrosis is one of the major causes of poor remodelling in HF after MI, thus prevention and reversal of cardiac fibrosis are essential for the treatment of CVD (González et al., 2018). Increasing studies on the structural domains of ncRNAs suggest that the important roles of various miRNAs in cardiac fibrosis after MI are realized by regulating the TGF-β/Smad signaling pathway. The miR-130a is downregulated in infarcted myocardium and hypoxia-induced cardiac fibroblasts, and its over-expression can reduce the size of the infarcted region and attenuate the cardiac impairment. This action mechanism may be related to the regulation of the activity of TGF-β/Smad signaling and the inhibition of the conversion of cardiac fibroblasts to myofibroblasts by the direct targeting of TGFβR1 (Feng et al., 2022). Similarly, upregulation of miR-328 was evident in MI mouse hearts and cardiac fibroblasts, and knockdown of miR-328 inhibited cardiac fibrosis in mice after MI. Moreover, TGFβRIII serves as a direct target of miR-328, and miR-328 over-expression can target and inhibit TGFβRIII, thereby indirectly activating the TGF-β1 signaling pathway, and promoting the collagen production (Du et al., 2016). Similarly, miR-21 expression was upregulated in MI mouse myocardial tissue and AngII-treated cardiac fibroblasts, and knockdown of miR-21 partially inhibited the expression of TGF-β and Smad2/3. Furthermore, Spry1 is one of the target genes of miR-21, and inhibition of its expression reverses the effect of knockdown on TGF-β signaling and promoted the myofibroblast transformation (Li et al., 2020a). Therefore, by targeting and regulating the miR-21-Spry1 axis, new insights into the treatment of cardiac fibrosis after MI can be provided. As exhibited in recent clinical research, miR-205-3p expressions were greatly downregulated in the plasma of MI patients, and miR-205-3p over-expression obviously inhibited the TGF-β1-induced cardiac fibroblast fibrosis (Qiao et al., 2021).

Cardiac fibrosis plays an important role in progression of various CVDs, including MI, atrial fibrillation (AF), and pressure overload-induced cardiac hypertrophy. In addition, it may lead to the reduced cardiac diastolic and systolic function later, possibly developing into HF finally. Some miRNAs can perfect cardiac function by modulating the TGF-β/Smad signaling pathway, thereby improving pressure-load-induced cardiac fibrosis. According to Zhang et al. (Zhang et al., 2018a), miR-323a-3p was discovered to be upregulated in the transverse aortic constriction (TAC)-induced cardiac tissues and the Ang II-treated rat cardiac fibroblasts. Besides, over-expression greatly increased the expressions of Col-I, Col-III, TGF-β, matrix metallopeptidase (MMP) 2, and MMP9 and enhanced the cardiac fibrosis by targeting the induction of the TIMP3-TGF-β pathway. Moreover, miR-26a expression was reduced in plasma and myocardial tissue of hypertensive cardiac fibrosis rats. While, transfection of miR-26a mimics significantly attenuate the expressions of Col-I, Col-III, MMP2, and TGFβRI in the AngII-induced cardiac fibroblasts, indicating that miR-26a inhibited the proliferation of cardiac fibroblasts. Besides, enhancer of zeste homolog 2 (EZH2), connective tissue growth factor (CTGF), and Smad4 are proven to exist at binding sites to miR-26a. The miR-26a can directly target CTGF and Smad4, thereby reducing collagen production and ECM deposition, and inhibiting cardiac fibroblasts proliferation *via* the EZH2/p21 pathway as well (Zhang et al., 2020a). Similarly, miR-29b is lowly expressed in both AngII-induced fibrotic hearts and cardiac fibroblasts, while the over-expression of miR-29b can prevent AngII-induced cardiac fibrosis and cardiac dysfunction by blocking the TGF-β/Smad3 signaling pathway, with the therapeutic potential for hypertensive heart disease (Zhang et al., 2014).

EndMT is a cellular transdifferentiation program, in which endothelial cells partially lose their endothelial properties and acquire mesenchymal-like characteristics, thereby promoting the development of cardiac fibrosis. Partial miRNAs improve the endothelial dysfunction and inhibit EndMT by blocking the TGF-β/Smad signaling pathway, which makes for the inhibition of cardiac fibrosis development (Ding et al., 2021). According to Ding et al., the miR-195-5p expression is upregulated in DCM rat myocardium and HG-induced human umbilical vein endothelial cells (HUVEC), and the inhibition of miR-195-5p reduces the EndMT in DCM rats and HG-induced EndMT in HUVEC. Moreover, Smad7, a negative regulator of TGF-β1 signaling, acts as a direct target of miR-195-5p, and can mediate the process of cardiac fibrosis in DCM. The silencing miR-195-5p and the promoting smad7 expression can inhibit the activation of TGF-β1/smad signaling pathway, thereby blocking EndMT and attenuating cardiac fibrosis in DCM (Meng et al., 2019; Ding et al., 2021). Similar to the researches above, miR-21 expression was upregulated in the myocardium of Type I diabetic (T1DM) mice, and the inhibition of miR-21 expression can suppress the expression of inhibitory fibrosis markers and alleviate the EndMT in the hearts of T1DM mice. Besides, as indicated by the Western blotting, Smad7 is obviously downregulated in T1DM mice, while p-Smad2 and p-Smad3 are greatly upregulated. Moreover, by inhibiting miR-21 and up-regulating Smad7 expression, the activation of p-Smad2 and p-Smad3 pathways in the hearts of T1DM mice could be blocked, thereby inhibiting EndMT activation and cardiac fibrosis (Li et al., 2020b).

AF is a common clinical arrhythmia. Cardiac fibrosis, as one of the pathogeneses of AF, seriously affects the progression of AF. Simultaneously, as prove by numerous evidences, miRNAs can activate and inhibit the development of cardiac fibrosis by blocking the TGF-β/Smad signaling pathway (Lai et al., 2022). For example, miR-10a inhibits AF-induced cardiac fibrosis by blocking the TGF-β1/Smads signaling pathway, thereby reducing the proliferation of cardiac fibroblasts, inhibiting the collagen formation, and reducing the atrial structural remodelling (Li et al., 2019a). The miR-29b is lowly expressed in AF rat atrial tissues. By promoting its expression, atrial fibrosis and AF in rats can be attenuated, which may be related to miR-29b by targeting TGFβRI and inhibiting the activation of the Smad2/3 signaling pathway (Han et al., 2022). Furthermore, miR-135b is lowly expressed in AF patients, isoproterenol (ISO)-induced rat models, and *in vitro* rat cardiac fibroblasts (RCFs). Whereas, TGF-βR1, TGF-βR2, and Smad2 are upregulated as their target gene expression. By promoting miR-135b expression, it can inhibit TGF-βR1, TGF-βR2, and Smad2 expression. These findings indicated that miR-135b exerted antiatrial fibrosis effects *in vitro* and *in vivo* by the inhibition of the TGF-β/Smads signaling pathway, thus providing a potential approach for the prevention and treatment of human AF (Wang et al., 2021a).

TGF-β induces collagen deposition in the ECM, and some miRNAs inhibit collagen production by regulating the TGF-β/Smad signaling pathway, thereby inhibiting ECM deposition, and alleviating cardiac fibrosis. Xiao et al. (Xiao et al., 2021) discovered that miR-1202 promoted the proliferation, differentiation, and collagen synthesis of HCFs by activating the TGF-β1/Smad2/3 signaling pathway, and downregulated the nNOS by binding to target sites in its mRNA. While, nNOS protected HCFs from TGF-β1-induced differentiation and collagen synthesis *via* the TGF-β1/Smad2/3 pathway. Thus, transfection of miR-1202 significantly increased the Col-I, Col-III, and *a*-SMA expressions as well as the Smad2/3 phosphorylation levels, thereby increasing ECM deposition. Furthermore, miR-29a is closely associated with cardiac fibrosis in HCM patients. Activation of ET1 signaling in cardiomyocytes increases ROS production and stimulates TGFβ expression, while miR-29a inhibits TGFβ-induced elastin (ELN) and collagen expression in cardiac fibroblasts, thereby suppressing the process of cardiac fibrosis (Roncarati et al., 2014).

TGF-β1 is an important regulator in the development of tissue fibrosis, and some miRNAs can inhibit the development of cardiac fibrosis by suppressing the expression of TGF-β1 (Stawowy et al., 2004). The protein levels of αSMA, Col-I, and POSTN in mouse heart tissue and the secreted collagen levels in cell culture supernatants increase obviously in response to TGFβ1 stimulation. In contrast, over-expression of miR-675 could reverse the TGFβ1-induced remodelling and the proliferation of mouse cardiac fibroblasts by targeting TGFβR1 (Wang et al., 2019a). Similarly, there were negative correlation between miRNA-663 and TGF-β1 expression in endomyocardial myocardial biopsies from patients with cardiac fibrosis, while over-expression of miRNA-663 resulted in the decreased expression of the cardiac fibrosis markers plasminogen activator inhibitor-1 (PAI-1) and tissue inhibitor of metalloproteinase-1 (TIMP-1) (Wu et al., 2019). MiR-30c expression is downregulated in the atrial samples from AAC model rats and in the TGF-β1-stimulated RCFs, whereas miR-30c over-expression inhibited TGF-β1-induces proliferation, differentiation, migration, and ECM synthesis in cardiac fibroblasts, by directly targeting TGFβRII (Xu et al., 2018). Besides, a high NaCl diet can also induce cardiac fibrosis, low miR-210-5p expression was observed in NaCl-added rat cardiac fibroblasts (NRCFs), while over-expression miR-210-5p reversed the upregulated levels of collagen I, *a*-SMA, and TGF-β1 in NRCFs by targeting TGFβR1, thereby attenuating cardiac fibroblasts activation and ameliorating cardiac fibrosis (Zhao et al., 2021).

Some miRNAs promote the development of cardiac fibrosis, by activating the TGF-β/Smad signaling pathway. Zhang et al. (2022a) verified that there was a positive correlation between miR-208b and miR-21 and the expressions of TGF-β1 and Smad3. The miR-208b/miR-21 promoted the development of cardiac fibrosis by activating the TGF-β1/Smad3 signaling pathway. Furthermore, in HCFs, miR-216a was able to activate the TGF-βRI/Smad2 signaling pathway, thereby reducing the collagen production and the *a*-SMA protein expression in the activated HSC through negative regulation of Smad7 expression (Tao et al., 2019).

Cardiac fibrosis is one of the major pathological features of diabetic cardiomyopathy. Yang and Zhao, (2022) presented that miR-30a-5p expression was downregulated in myocardial tissue and HG-treated cardiac fibroblasts of diabetes mellitus (DM) rats. Moreover, miR-30a-5p over-expression reduced Smad2 levels, inhibited collagen formation in HG-stimulated cardiac fibroblasts and DM rats. In addition, decreased HG induced proliferation of cardiac fibroblasts, thereby lowering the collagen deposition and thus delaying the progression of cardiac fibrosis in diabetic cardiomyopathy.

PAH pathology involves ECM remodeling in cardiac tissue, which accelerates the progression of cardiac fibrosis. Connolly et al. reported that miR-1-5p and TGF-βR1 were inversely expressed in the RV of MCT-treated rats, and transfection with miR-1-5p mimics significantly lowered the TGF-βR1 *in vitro*, It suggests that miR-1 causes cardiac hypertrophy by targeting TGF-βR1 and reducing TGF-β signaling, thereby resulting in cardiac hypertrophy [ 86].

### 3.3 Roles of miRNAs in regulating other non-classical signaling pathways in cardiac fibrosis

In addition to regulating the classical signaling pathways such as TGF-β/Smad in cardiac fibrosis, miRNAs can also participate in the cardiac fibrosis by mediating some non-classical signaling pathways. For example, HG greatly increased the expression of TGFβ1, IL-1β factors, and phosphorylated NF-κB activity. Smad7, a target of miR-150-5p, whose expression is upregulated, inhibits the NF-κB activity and IL-1β production, and suppresses the progression of cardiac fibrosis by inhibiting the TGF-β1/Smad signaling pathway. Thus, it can inhibit TGF-β1/Smad-induced cardiac fibrosis and NF-κB-associated cardiac inflammation by silencing miR-150-5p and up-regulating Smad7 expression, thereby ameliorating the HG-induced cardiac fibroblasts injury (Che et al., 2020a).

In addition, MAPK (ERK1/2, p38MAPK, and JNK) signaling pathways are activated during cardiac fibrosis. The miR-327 expression is investigated to be upregulated in TAC-induced cardiac fibrotic tissue and Ang II-induced cardiac fibroblasts, and the inhibition of miR-327 expression restrains the TAC-induced ERK1/2, p38MAPK, and JNK phosphorylation levels, thereby attenuating the cardiac fibrosis (Ji et al., 2018). Similarly, miR-143-3p is highly expressed in human and animal myocardial infarcted hearts and in TGFβ1-induced human cardiac fibroblasts. SPRY3 is a target gene of miR-143-3p, its downstream ERK, JNK, and p38MAPK signaling pathways are involved in the process of cardiac fibrosis, and the silencing SPRY3 promotes ECM by regulating the MAPK pathway over-accumulation. Thus, miR-143-3p directly targets SPRY3 by activating the p38MAPK, ERK, and JNK signaling pathways, which in turn regulates the biological functions of HCFs such as proliferation, migration, and transformation (Li et al., 2019b). The miR-223 is highly expressed in activated fibroblasts, and promotes their proliferation, migration, and differentiation. While the silencing miR-223 inhibits TGF- β1-induced expression of collagen I, collagen III, and *a*-SMA proteins, and the over-expression of miR-223 and downregulation of its target recombinant Ras GTPase activating protein 1 (RASA1) promote the phosphorylation of MEK1/2, ERK1/2, and AKT in cardiac fibroblasts, indicating that miR-223 can promote proliferation, migration, and differentiation of cardiac fibroblasts by down-regulating RASA1 expression and activating the renin-angiotensin system (RAS) signaling pathway, thereby aggravating cardiac fibrosis after MI (Liu et al., 2018a). Similarly, miR-378 over-expression inhibited cardiac fibrosis during the cardiac adaptation phase of pressure overload, and restrained the phosphorylation of p38MAPK and Smad2/3 activated by mechanical overload. Moreover, miR-378 inhibited the p38MAPK and Smad2/3 signaling pathways by directly targeting MAPK kinase 6 (MKK6), thereby suppressing the pressure overload-induced cardiac Fibrosis (Yuan et al., 2018). The miR-1468-3p increasing in both the senescent healthy hearts and the patients with sudden cardiac death suffering from primary cardiac fibrosis can enhance the cellular senescence and collagen deposition by promoting TGF-β1-p38 signaling and exerting a dual role (Lin et al., 2020).

Furthermore, the extent of fibrosis depends on the balance between pro-fibrotic and pro-inflammatory signaling. NF-κB is a major determinant of the inflammatory process, and it can promote the transcription of several pro-inflammatory genes, including TNF-α, IL-1β, and IL-6. Adriamycin induces ROS production, and triggers the TGF-β pathway, thereby activating the conversion of cardiac fibroblasts to myofibroblasts. While, using Ant34a (miR-34a inhibitor) in the treatment can greatly reduce NF-κB, IL-6, and TNF-α levels in the hearts of adriamycin-induced animals, and then decrease the Col-I expression and collagen deposition (Piegari et al., 2020) (Table 1).

**TABLE 1:** Mechanism of miRNA regulating TGF signaling pathway.

Type	ncRNA	Sample sources	Dysregulation in MF	Pathway	Effects	References
miRNA	miR-130a	Infarcted myocardium and hypoxic CFs of mouse	Downregulated	TGFβR1、TGF-β/Smad	Inhibition the conversion of CFs to myofibroblasts	Feng et al. (2022)
miRNA	miR-328	MI mouse hearts and CFs	Upregulated	TGFβRIII、TGF-β1	Promoting the collagen production	Du et al. (2016)
miRNA	miR-21	MI mouse myocardial tissue and AngII-treated CFs	Upregulated	TGF-β、Smad2/3、Spry1	Inhibition the conversion of CFs to myofibroblasts	Li et al. (2020a)
miRNA	miR-205-3p	MI patients	Downregulated	TGF-β1	Inhibited the TGF-β1-induced cardiac fibroblast fibrosis	Qiao et al. (2021)
miRNA	miR-323a-3p	The transverse aortic constriction (TAC)-induced cardiac tissues and the Ang II-treated rat CFs	Upregulated	TGF-β、MMP2 、MMP9	Enhanced the cardiac fibrosis	Zhang et al. (2018a)
miRNA	miR-26a	Plasma and myocardial tissue of hypertensive MF rats	Downregulated	EZH2/p21 pathway、CTGF、 Smad4	Reducing collagen production and ECM deposition, and inhibiting CFs proliferation	Zhang et al. (2020a)
miRNA	miR-29b	AngII-induced fibrotic hearts and CFs	Downregulated	TGF-β/Smad3	Prevent AngII-induced cardiac fibrosis and cardiac dysfunction	Zhang et al. (2014)
		AF rat atrial tissues	Downregulated	TGFβRI、Smad2/3	Atrial fibrosis and AF in rats can be attenuated	Han et al. (2022)
miRNA	miR-195-5p	DCM rat myocardium and HG-induced human umbilical vein endothelial cells	Upregulated	TGF-β/Smad	Mediate the process of cardiac fibrosis in DCM	Ding et al. (2021)
miRNA	miR-21	the myocardium of Type I diabetic (T1DM) mice	Upregulated	Smad2/3、Smad7	Inhibiting EndMT activation and MF	Li et al. (2020b)
miRNA	miR-10a	AF rat models and CFs	Upregulated	TGF-β1/Smads	Inhibiting the collagen formation and reduce atrial structural remodelling	Li et al. (2019a)
miRNA	miR-135b	AF patients, isoproterenol (ISO)-induced rat models and rat cardiac fibroblasts	Downregulated	TGF-βR1、TGF-βR2 、 Smad2	Anti-atrial fibrosis	Wang et al. (2021a)
miRNA	miR-1202	human CFs	Upregulated	TGF-β1/Smad2/3	Activating CFs proliferation, differentiation, and collagen synthesis	Xiao et al. (2021)
miRNA	miR-29a	HCM patients	Upregulated	TGFβ、ELN	Suppressing the process of cardiac fibrosis	Roncarati et al. (2014)
miRNA	miR-675	Mouse myocardial tissue and CFs	Downregulated	TGFβR1	Remodelling and the proliferation of mouse CFs	Wang et al. (2019a)
miRNA	miRNA-663	Endomyocardial myocardial biopsies from patients with MF	Downregulated	TGF-β1	Inhibit myocardial fibrosis	Wu et al. (2019)
miRNA	miR-30c	AAC model rats and in the TGF-β1-stimulated rat CFs	Downregulated	TGFβRII、TGF-β1	Inhibited TGF-β1-induced proliferation, differentiation, migration, and ECM synthesis in CFs	Xu et al. (2018)
miRNA	miR-210-5p	NRCFs	Downregulated	TGFβR1	Attenuating CFs activation and ameliorating cardiac fibrosis	Zhao et al. (2021)
miRNA	miR-208b and miR-21	AMI patients	Upregulated	TGF-β1/Smad-3	Promotes the development of cardiac fibrosis	Zhang et al. (2022a)
miRNA	miR-216a	HCFs	Upregulated	TGF-βRI、Smad2、Smad7	Reduced collagen production and the *a*-SMA protein expression	Tao et al. (2019)
miRNA	miR-30a-5p	Myocardial tissue and HG-treated CFs of DM rats	Downregulated	Smad2	Delaying the progression of MF in diabetic cardiomyopathy	Yang and Zhao (2022)
miRNA	miR-150-5p	High glucose-treated cardiac fibroblasts	Upregulated	TGFβ1、IL-1β、NF-κB	Ameliorating HG-induced CFs injury	Che et al. (2020a)
miRNA	miR-327	TAC-induced cardiac fibrotic tissue and Ang II-induced cardiac fibroblasts	Upregulated	ERK1/2、p38MAPK、JNK	Attenuating TAC-induced cardiac fibrosis	Ji et al. (2018)
miRNA	miR-143-3p	Human and animal myocardial infarcted hearts	Upregulated	ERK、JNK、p38MAPK	Regulates the biological functions of HCFs such as proliferation, migration, and transformation	Li et al. (2019b)
miRNA	miR-223	Activated fibroblasts	Upregulated	RASA1、MEK1/2、ERK1/2、AKT	Aggravating MF after MI	Liu et al. (2018a)
miRNA	miR-378	Mouse TAC models	Downregulated	MKK6、p38MAPK 、 Smad2/3	Uppressing the pressure overload-induced cardiac Fibrosis	Yuan et al. (2018)
miRNA	miR-1468-3p	Senescent healthy hearts and the patients with sudden cardiac death suffering from primary MF	Upregulated	TGF-β1、p38	Enhance the cellular senescence and collagen deposition	Lin et al. (2020)
miRNA	miR-34a	Rat model of doxorubicin toxicity	Upregulated	NF-κB、IL-6、TNF-α	Reduce the collagen deposition	Piegari et al. (2020)
miRNA	miR-1-5p	PAH rat	Downregulated	TGF-βR1	Inhibition of PAH-induced cardiac fibrosis	Connolly et al. (2020)
miRNA	miR-325-3p	PAH rat	Upregulated	MMP2/9、HE4	Inhibited the fibrosis of cardiac fibroblasts	Tang et al. (2022)
miRNA	miR-1	PAH rat	Upregulated	collagen I、collagen III、α-SMA、CTGF、PI3K、AKT	Inhibit right ventricular hypertrophy and fibrosis	Liu et al. (2021a)

PAH usually causes right ventricular dysfunction, which is closely associated with cardiac fibrosis. Increased expression of type I and III collagen and MMP2/9 in the PAH group, as reported by Tang et al. (2022) suggested that PAH induced myocardial fibrosis in rats, whereas miR-325-3p overexpression attenuated myocardial fibrosis in PAH rats. In addition, dual-luciferase and bioinformatics reported that miR-325-3p exhibited binding sites to human epididymis protein 4 (HE4), and miR- 325-3p inhibited cardiac fibroblast fibrosis by targeting and regulating HE4. In addition, miR-1 was reported to exhibit an increased expression in the hypoxia-induced RV of PAH model rats, inhibiting the expressions of collagen I, collagen III, *a*-SMA, and CTGF, the further study revealed that expressions of p-phosphatidylinositol 3-kinase (PI3K) and p-AKT are upregulated in hypoxia-induced cardiac fibroblasts and are reversed by the transfected miR-1 antagomiR (Liu et al., 2021a). It means that miR-1 involves in the PAH-induced right ventricular hypertrophy and fibrosis by regulating the PI3K/AKT signaling pathway.

### 3.4 Regulation of TGF-β/Smad signaling pathway by lncRNAs in cardiac fibrosis

Dysregulation of LncRNAs and TGF-β is an important trigger of cardiac fibrosis. In recent decades, their link has been investigated and has exhibited that lncRNAs and TGF-β can interact in several mechanisms of cardiac fibrosis onset and progression. For example, as indicated by the research of Guo et al. (2022), the over-expression of lncRNA H19 which was a competing endogenous RNA for miR-29a/b-3p, greatly promoting the cardiac fibroblasts proliferation and collagen production. In contrast, the over-expression of miR-29a/b-3p could reverse the effects of H19 on cardiac fibroblasts activity and collagen production by targeting vascular endothelial growth factor A (VEGFA) and TGF-β. Moreover, the over-expression of lncRNA HOXA11-AS promoted the expression of TGFβ1 signaling pathway and downstream proteins, and enhanced the proliferation and migration of cardiac fibroblasts by enhancing the TGFβ1 activity, thereby promoting the progression of cardiac fibrosis (Wang et al., 2019b). Besides, expression of lncRNA FAF was downregulated in both Ang-induced heart tissue and cardiac fibroblasts of rat, and elevating its expression can inhibit the phosphorylation levels of Smad2/3 (Sun et al., 2020). It suggests that the over-expression of FAF inhibited the proliferation and transformation of fibroblasts and then relieve the collagen production and cardiac fibrosis. In another study, expressions of lncRNA MHRT were found to be elevated in TGF-β1-treated mouse heart tissues and cardiac fibroblasts. Over-expression of MHRT promoted the collagen production and proliferation of cardiac fibroblasts and reduced the expression of miR-3185. In addition, siMHRT could reverse the inhibitory effect of TGF-β1 on miR- 3185 expression. Thus, MHRT aggravated the myocardial collagen deposition by regulating miR-3185 expression, thereby exacerbating the cardiac fibrosis after MI (Lang et al., 2021).

TGF-β3 is a member of multifunctional peptide superfamily, regulates cell growth and differentiation, and exerts a special role in tissue fibrosis (Liu et al., 2022b). For example, Liu et al. reported that Vgll3 could positively regulate the expression of the key pro-fibrotic factor TGF-β3 and the downstream factor smad2/4. As demonstrated by dual luciferase reporter and Western blotting experiments, Vgll3 overexpression could promote the activation and proliferation of cardiac fibroblasts in mice. Such phenomenon was counteracted by TGF-β3, suggesting that Vgll3, a member of lncRNA, could promote the progression of cardiac fibrosis by activating the TGF-β3-related pathways (Stawowy et al., 2004). Being different from the above studies, lncRNA nuclear enriched abundant transcript 1 (NEAT1) is closely associated with the progression of lung, liver, and kidney fibrosis (Wang et al., 2019a; Wang et al., 2021b; Zhao et al., 2022). As reported, NEAT1 was significantly expressed in both HF patients and TAC-induced HF mouse models, inhibition of its expression attenuated the TGF-β1-induced cardiac fibrosis, GSK126 (an EZH2 inhibitor) weakened TGF-β1-induced downregulation of Smad7 expression and upregulation of p-Smad2/3, and upregulation of Smad7 reversed the cardiac fibrosis caused by NEAT1 over-expression (Zhang et al., 2020b). The lncRNA Cfast is highly expressed in heart tissue of MI mouse, and Cfast competitively interacts with COTL1 to prevent COTL1 from binding to TRAP1, thereby enhancing the formation of the TRAP1/Smad2/Smad4 complex and thus activating the TGF-β signaling pathway (Zhang et al., 2020c). Therefore, inhibiting the Cfast in the heart can prevent the pathological fibrotic remodelling and improve the cardiac function. LncRNA CRNDE is an RNA specifically expressed in heart tissues of human and mouse, and its over-expression inhibits the differentiation of cardiac fibroblasts to myofibroblasts, which may be related to the blocking of transcriptional activation of the TGF-β/Smad3 signaling pathway (Zheng et al., 2019). Moreover, LncRNA mannose-inhibitable adhesin-T7 receptor (MIAT) is upregulated in cardiac tissues of the MI mouse model, and knockdown of MIAT inhibits the collagen production and cardiac fibroblasts proliferation, thereby suppressing interstitial fibrosis by a mechanism related to MIAT sponging miR-24, and thus regulating Furin/TGF-β1 expression (Qu et al., 2017). Similarly, LncRNA MIAT attenuates its inhibitory effect on IL-17 by sponging miR-22-3p, thus leading to cardiac fibrosis (Qu et al., 2017). lncRNA MALAT1 expression is upregulated in diabetic mice and HG-induced fibroblasts, and acts as a sponge for miR-141, thereby promoting the upregulation of TGF-β1, p-Smad2, p Smad3, NLRP3, caspase-1, IL-18, and IL-1β. While over-expression of miR-141 can reduce the expressions of the above factors (Che et al., 2020b).

MI can induce excessive secretion of ECM by cardiac fibroblasts, and activate and remodel myocardium, thus leading to cardiac fibrosis and cardiac dysfunction (Travers et al., 2016). According to Luo et al. (2020), lncRNA 554 was upregulated in both cytoplasm and nucleus of the MI-induced cardiac fibroblasts, while the silencing 554 significantly downregulated the expression of TGF-β1 and Smad3. Such findings suggest that the inhibition of lncRNA 554 expression attenuates the cardiac fibrosis and enhance the cardiac function in MI mice. Similarly, lncRNA Ang362 was highly expressed in MI-induced cardiac fibrosis rats and RCFs, and promoted the TGF-β1-induced collagen expression by suppressing Smad7 expression, thereby aggravating the cardiac fibrosis (Chen et al., 2020a) (Table 2).

**TABLE 2:** Mechanism of lncRNA regulating TGF signaling pathway.

Type	ncRNA	Sample sources	Dysregulation in MF	Pathway	Effects	References
lncRNA	lncRNA H19	AF patients and CFs	Downregulated	miR-29a/b-3p、VEGFA、TGF-β	Inhibit CFs proliferation and collagen production	Guo et al. (2022)
		Fibrous tissue and activated CFs in heart	Upregulated	DUSP5、ERK1/2	Contributes to cardiac fibroblast proliferation and fibrosis	Tao et al. (2016)
lncRNA	lncRNA HOXA11-AS	Mouse CFs	Upregulated	TGFβ1	Enhanced the proliferation and migration of CFs by increasing TGFβ1 activity	Wang et al. (2019b)
lncRNA	lncRNA FAF	Ang-induced rat heart tissue and CFs	Downregulated	Smad2/3	Inhibiting collagen production and cardiac fibrosis	Sun et al. (2020)
lncRNA	lncRNA MHRT	TGF-β1-treated mouse heart tissues and CFs	Upregulated	miR-3185	Enhancing MF after MI	Lang et al. (2021)
lncRNA	LncRNA Vgll3	Mouse CFs	Upregulated	TGF-β3、Smad2、Smad4	Accelerating the development of cardiac fibrosis	Liu et al. (2022a)
lncRNA	lncRNA NEAT1	HF patients and TAC-induced HF mouse models	Upregulated	Smad2/3、smad7	Reversed the cardiac fibrosis	Zhang et al. (2020b)
lncRNA	lncRNA Cfast	MI mouse heart tissue	Upregulated	COTL1、TRAP1、Smad2/Smad4	Protected from pathological fibrotic remodelling and improved the cardiac function	Zhang et al. (2020c)
lncRNA	LncRNA CRNDE	Human and mouse heart tissue	Upregulated	TGF-β/Smad3	Inhibits the differentiation of cardiac fibroblasts to myofibroblasts	Zheng et al. (2019)
lncRNA	LncRNA MIAT	MI mouse model	Upregulated	miR-24、Furin、TGF-β1	Inhibits the collagen production and CFs proliferation	Qi et al. (2020)
lncRNA	LncRNA MALAT1	Diabetic mice and HG-induced fibroblasts	Upregulated	miR-141、Smad2/3、NLRP3、caspase-1、IL-18、IL-1β	Inhibit cardiac fibrosis	Che et al. (2020b)
lncRNA	lncRNA 554	Cytoplasm and nucleus of the MI-induced cardiac fibroblasts	Upregulated	GF-β1、Smad3	Attenuated MF and improved cardiac function in MI mice	Luo et al. (2020)
lncRNA	lncRNA Ang362	MI-induced cardiac fibrosis rats and CFs	Upregulated	Smad7、TGF-β1	accelerating the development of cardiac fibrosis	Chen et al. (2020a)
lncRNA	LINC00961	Injury and EndMT in HCMECs induced by TGF-β	Downregulated	PTEN、PI3K、AKT、mTOR	Inhibit endothelial interstitial transformation and myocardial fibrosis	Hu et al. (2022)
lncRNA	lncRNA CFAR	MI-induced fibrotic mouse model and TGF-β-induced fibrotic cell model	Upregulated	miR-449a-5p、LOXL3、mTOR	Promote cardiac fibrosis	Zhang et al. (2022b)
lncRNA	lncRNA GAS5	Fibrous tissue and activated CFs in heart	Downregulated	miR-21、PTEN、MMP2	Inhibit cardiac fibrosis	Tao et al. (2017)
lncRNA	Linc00092	Heart tissue and heart CFs	Downregulated	TGFβ1、ERK	Attenuate HCF activation by suppressing glycolysis	Chen et al. (2020b)
lncRNA	lncRNA-HCG18	HCFs	Upregulated	miR-133a、ERK、EGFR	Inhibit the cell proliferation of cardiac fibroblasts	Shi et al. (2022)
lncRNA	LINC00636	CFs and exosomes isolated from the PF of nAF patients	Upregulated	miR-450a-2-3p、MAPK1	Improve cardiac fibrosis in patients with atrial fibrillation	Liu et al. (2021b)

### 3.5 LncRNA regulates other non-classical signaling pathways in cardiac fibrosis

Similar to the role of miRNAs, LncRNAs involves in the progression of cardiac fibrosis by regulating PI3K-Akt, MAPK, and other signaling pathways in addition to the classical signaling pathway of TGF-β. For example, EndMT plays an important role in pathophysiological processes such as myocardial ischemia-reperfusion, MI, diabetic cardiomyopathy, and fibrosis. There is an example: EndMT plays an important role in pathophysiological processes such as myocardial ischemia-reperfusion, myocardial infarction, diabetic cardiomyopathy, and fibrosis; and TGF-β-induced EndMT can be attenuated by knocking down the LINC00961 by a mechanism related to activation of phosphatase and tensin homolog (PTEN) expression and inhibition of PI3K, AKT, and mammalian target of rapamycin (mTOR) (Hu et al., 2022). mTOR, as a PI3K- Akt downstream gene, mainly regulates the cardiac fibrosis by affecting the expressions of pro-fibrotic factors. A study revealed that lncRNA CFAR expression is upregulated in MI-induced fibrosis mouse models and TGF-β-induced fibrotic cell models. The mechanism analysis suggested that lncRNA CFAR, a ceRNA of miR-449a-5p, promotes the Lysyl oxidase-like protein 3 (LOXL3) expression, and overexpression of which may elevate the mTOR expression, aggravating the cardiac fibrosis (Zhang et al., 2022b). Additional evidence suggests that PTEN is a major regulator of the PI3K/Akt pathway, and inhibited PTEN promotes the ECM deposition and myocardial fibrosis in mice (Yuan et al., 2019). LncRNA GAS5 is lowly expressed in cardiac fibrotic tissues and activated cardiac fibroblasts, and its overexpression can inhibit the cardiac fibroblast proliferation. In addition, miR-21 can play a pro-fibrotic role in multiple organs, and lncRNA GAS5 can act as a sponge for miR-21 to inhibit the PTEN/MMP2 expression and the progression of cardiac fibrosis (Tao et al., 2017).

The ERK1/2 signaling pathway, a family member of MAPK and in the downstream of TGF-β1, can be activated during the progression of cardiac fibrosis (Kong et al., 2014). Linc00092, a glycolysis-related lncRNA and expressed mainly in cardiac tissues and cardiac fibroblasts, inhibits the TGFβ1-induced HCF activation by suppressing glycolysis, and its overexpression significantly and consistently attenuates the phosphorylation of ERK and subsequently inhibits the ERK activation and HCF activation (Chen et al., 2020b). Shi et al. (2022) showed that silencing lncRNA-HCG18 in cardiac fibroblasts significantly promoted the hsa-miR-133a expression, inactivated the ERK1/2 pathway and inhibited the cell proliferation of cardiac fibroblasts by downregulating the EGFR expression. It suggests that lncRNA-HCG18 can exert an regulatory effect in progression of cardiac fibrosis through the hsa-miR-133a/ERK/EGFR axis. Similarly, lncRNA H19 expression is upregulated in cardiac fibrotic tissues and activated fibroblasts, while overexpression of DUSP5 abrogates the pro-proliferative effect of H19 in cardiac fibroblasts by a mechanism associated with inhibition of ERK1/2 phosphorylation (Tao et al., 2016). In addition, MAPK1 has been reported to involve in various biological processes including myocardial fibrosis, and can be inhibited by exosomes containing LINC00636 by elevating the miR-450a-2-3p expression, thus alleviating the cardiac fibrosis in patients with AF (Liu et al., 2021b).

### 3.6 Regulation of TGF-β/Smad signaling pathway by CircRNA in cardiac fibrosis

The main feature of cardiac fibrosis is the activation, proliferation, and transformation of cardiac fibroblasts. Some circRNAs can regulate the miRNAs expression and the TGF-β/Smad signaling pathway, thus playing a key role in the pathogenesis of cardiac fibrosis. According to Li et al. (2020c), circRNA heterogeneous nuclear ribonucleoprotein H1 (HNRNPH1) increased and mainly expressed in cardiac fibroblasts in heart after ischemia. Meanwhile, it limited the differentiation of cardiac fibroblasts into myofibroblasts by miR-216-5p-SMAD7-mediated degradation of TGF-β1, thus becoming a potential new target for the treatment of cardiac fibrosis after ischemia. In addition, CircRNA NFIB has been found to be lowly expressed in heart tissue of MI mouse and TGF-β-treated cardiac fibroblasts, and its expression inhibits the cardiac fibroblasts proliferation by a mechanism related to sponging miR-433 to upregulate the AZIN1 and JNK1 and inhibit the cardiac fibroblasts proliferation (Zhu et al., 2019).

Cardiac fibrosis leads to the expression of *a*-SMA by activated myofibroblasts, and promotes the formation of ECM proteins (COL1A1 and COL3A1). Some circRNAs can regulate TGF-β/Smad expression, and inhibit fibrotic proteins expression, thereby suppressing the onset and progression of cardiac fibrosis. For example, as presented by Ni et al. (2019), circRNA HIPK3 was highly expressed in AngII-treated cardiac fibroblasts and mouse heart tissue, and promoted cardiac fibroblasts proliferation, migration, and cardiac fibrosis by sponging miR-29b-3p and up-regulating a-SMA, COL1A1, and COL3A1 expressions. Similar to the above studies, circRNA HIPK3 expression is upregulated in hypoxia-induced cardiac fibroblasts, and silencing circHIPK3 inhibits the cell proliferation and migration. The bioinformatics and immunofluorescence reports indicate that circRNA HIPK3 can bind to miR-152-3p to inhibit the proliferation of cardiac fibroblasts and expressions of ColI, ColIII, and TGF-β2 after hypoxic stimulation. Therefore, circHIPK3 is a molecular regulator during the cardiac fibrosis through miR-152-3p/TGF-β2 signaling (Liu et al., 2020). Similarly, Zhou and Yu (2017) discovered that circRNA_010567 was greatly expressed in diabetic mouse myocardium and AngII-induced cardiac fibroblasts, and regulated the expression of TGF-β1 and fibrosis genes by acting as a sponge for miR-141.

**FIGURE 2:**
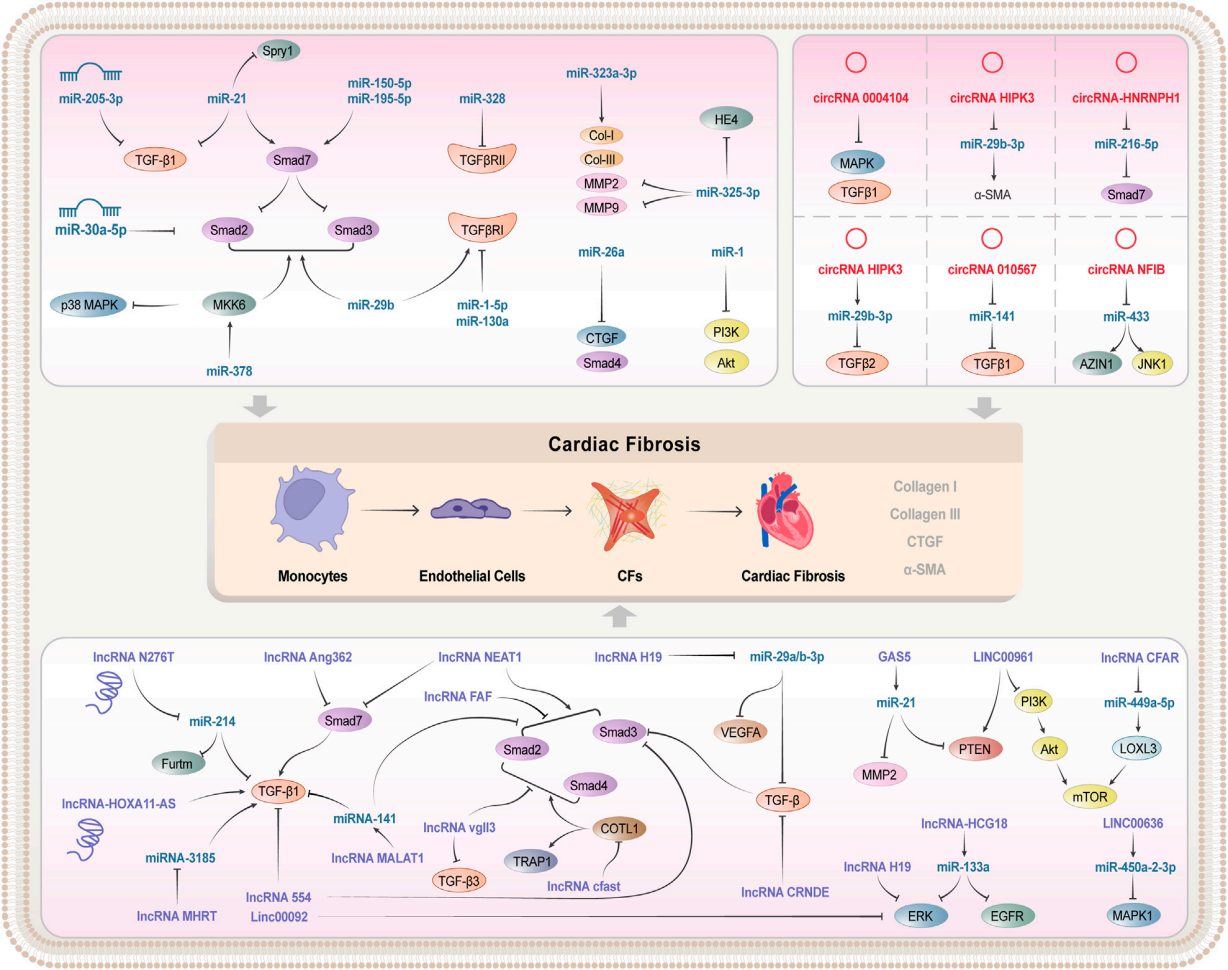
Mechanism of non-coding RNA regulating TGF-β/Smad signaling pathway.

Atrial fibrosis plays an important role in the progression of AF. The dysregulation of some circRNAs may promote AF, and thus becomes a potential regulator and biomarker of persistent AF. A clinical research indicated that circRNA_0004104 was significantly downregulated in the plasma of patients with persistent AF, and that it could negatively regulate the expression of MAPK and TGF-β1. This evidence suggests that circRNA_0004104 could promote cardiac fibrosis by targeting MAPK and TGF-β signaling pathways, and that elevating the circRNA_0004104 0004104 expressions can be a potential strategy to treat the f cardiac fibrosis and persistent AF (Gao et al., 2021). In the above section, we summarized the mechanism of different non-coding RNA regulating TGF signaling pathways in cardiac fibrosis, and they played an important role in the process of cardiac fibrosis (Table 3; Figure 2).

**TABLE 3:** Mechanism of lncRNA regulating TGF signaling pathway.

Type	ncRNA	Sample sources	Dysregulation in MF	Pathway	Effects	References
circRNA	circRNA HNRNPH1	Rat model of MI	Upregulated	miR-216-5p-SMAD7、TGF-β1	Limited the differentiation of CFs into myofibroblasts	Li et al. (2020c)
circRNA	circRNA NFIB	MI-induced mouse heart tissue and TGF-β-treated CFs	Downregulated	miR-433、AZIN1、JNK1	Inhibited the CFs proliferation	Zhu et al. (2019)
circRNA	circRNA HIPK3	AngII-treated CFs and mouse heart tissue	Upregulated	miR-29b-3p、a-SMA、COL1A1、COL3A1	Promoted CFs proliferation, migration, and cardiac fibrosis	Ni et al. (2019)
		Hypoxia-induced CFs	Upregulated	miR-152-3p、ColI、ColII、TGF-β2	Promote the transition of hypoxia-induced CFs to myofibroblasts	Liu et al. (2020)
circRNA	circRNA_010567	Diabetic mouse myocardium and AngII-induced CFs	Upregulated	miR-141、TGF-β1	Regulated the expression of TGF-β1 and fibrosis genes	Zhou and Yu (2017)
circRNA	circRNA_0004104	Persistent AF	Downregulated	MAPK、TGF-β1	Promote cardiac fibrosis	Gao et al. (2021)

## 4 Alternative therapies for TGF-β/Smad signaling pathway modulation in TCM against cardiac fibrosis

Traditional Chinese medicine (TCM) has been practiced in China for thousands of years, with widespread clinical applications gained. It has unique clinical advantages due to its multi-component, multi-target, multi-pathway characteristics, low side effects, and good ability to reduce drug resistance (Yu et al., 2022). In recent years, more and more studies have focused on the great potential exhibited by TCM in the prevention and treatment of cardiac fibrosis. In this work, the single, compound, and proprietary Chinese medicines, that can prevent and treat cardiac fibrosis by targeting and modulating the TGF-β/Smad signaling pathway, are summarized, with a view to providing new ideas and research directions for the development of new anti-fibrotic drugs.

### 4.1 Anti-cardiac fibrosis natural products

#### 4.1.1 Alkaloids

Neferine is a major bisbenzylisoquinoline alkaloid extracted from lotus seeds. It was found that Neferine can inhibit HG-induced expression of collagen I and III and TGF-β1, and restrain the migration of cardiac fibroblasts (Zhao et al., 2010). Moreover, Neferine can suppress the proliferation of cardiac fibroblasts and collagen synthesis by inhibiting TGF-β1-Smad, ERK, and p38MAPK signaling pathways, thus exerting the anti-fibrotic effects, and improving myocardial function in diabetic mice (Liu et al., 2016). Picrasidine, the main active ingredient extracted from bitter ginseng, was proven to be able to inhibit Smad2/3 phosphorylation levels and TGF-β1 expression in the diabetic and cardiomyopathic rats and cardiac fibroblasts cultured with high glucose. Therefore, it exerts the anti-fibrotic effects by restraining the activation of the TGF-β1/Smad signaling pathway, improving the cardiac compliance and cardiac function (Zhang et al., 2018b).

#### 4.1.2 Terpenoids

Zerumbone, a scopoleene sesquiterpene isolated from Syringa pinnatifolia, has anti-inflammatory and antioxidant effects. It inhibits the conversion of cardiac fibroblasts to myofibroblasts by restraining TGF-β1, p-Smad2/3, and MMP-2/9 and upregulating the expression of Smad7, thus protecting the myocardium from injury and preserving cardiac function (Li et al., 2022c). Ginkgolide B, a natural terpenoid derived from Ginkgo biloba, has anti-inflammatory, antioxidant, and anti-apoptotic effects (Wu et al., 2013). According to Jiang et al. (Jiang et al., 2020), ginkgolide B significantly reduced the expression of TGF-β1 and the phosphorylation level of Smad2/3 in diabetic rats, and also inhibited the expressions of Col-I and Col-III by suppressing the activation of ERK1/2, JNK, and p38MAPK signaling pathways, thereby restraining the development of diabetic cardiac fibrosis. *Centella asiatica* is a triterpenoid extracted from *Centella asiatica* with anti-inflammatory, antioxidant, and anti-fibrotic effects. *Centella asiatica* maintains the collagen metabolic homeostasis, and inhibits the development of cardiac fibrosis by restraining the expressions of Col-I, Col-III, CTGF, and PAI-1 in the left ventricle and AngII-induced cardiac fibroblasts of spontaneously hypertensive (SHR) rats. Besides, *centella asiatica* effectively inhibits Smad2/3 phosphorylation and up-regulates Smad7 expression in the hearts of SHRs, thereby suppressing cardiac fibroblasts proliferation and maintaining collagen metabolic homeostasis, and finally alleviating the overburden pressure-induced cardiac fibrosis (Lu et al., 2018). Betulinic acid, a pentacyclic triterpenoid isolated from birch bark, inhibits the proliferation and differentiation of cardiac fibroblasts induced by high glucose, and suppresses the expression of Col-I, Col-III, and fibronectin by reducing the phosphorylation level of Smad2/3 and TGF-β1 expression in cardiac fibroblasts, thereby relieving the hyperglycaemia-induced cardiac fibrosis (Jiang et al., 2017).

#### 4.1.3 Flavonoids

Soy glycosides and isoflavones exist in soy foods and soy oil, and have anti-inflammatory, antioxidant, and anti-apoptotic effects. They can inhibit the expressions of *a*-SMA, Col-I, and Col-III, thereby suppressing cardiac fibroblasts activation and ECM deposition. Moreover, they can restrain proliferation and migration of cardiac fibroblasts by reducing the phosphorylation levels of Smad2/3 and TGF-β1, suppressing the cardiac disease (Yu et al., 2020). Apigenin is a natural flavonoid compound existed in a variety of vegetables and plants in the form of glycosides, with anti-inflammatory, anti-tumour, and anti-fibrotic effects (Zhou et al., 2016). According to Wang et al. (Feng et al., 2021), Apigenin inhibited TGF-β1-stimulated differentiation and collagen synthesis of cardiac fibroblasts by increasing miR-122-5p expression, and exerted antifibrotic effects by downregulating the Smad2/3 expression and targeting HIF-1α, thereby upregulating the Smad7 expression. Glycyrrhizin is a flavanone compound derived from the root of Glycyrrhiza glabra. According to the previous researches, TGF-β1 and Smad2 were highly expressed in ISO-induced mouse heart tissue and AngII-induced cardiomyocytes, and glycyrrhizin relieved fibrosis in mice and cardiomyocytes by suppressing the activation of TGF-β1/Smad2 signaling pathway (Li et al., 2021b). Silymarin is an active compound extracted from the silymarin plant, and it can reduce TGF-β1 expression and Smad2/3 phosphorylation levels and increase Smad7 expression, thereby decreasing cardiac fibrosis and collagen deposition (Meng et al., 2019). Baicalin, a natural flavonoid present in the roots of Scutellaria baicalensis, can inhibit the Ang II-induced fibrosis in RCFs and the cardiac fibrosis caused by AAC-induced pressure overload, with a mechanism related to the inhibition of TGF-β over-expression, Smad2/3 hyperphosphorylation, and Smad4 upregulation (Xiao et al., 2018).

#### 4.1.4 Saponins

Astragaloside IV, one of the active components of Astragalus, inhibits the expression of *a*-SMA and ColI, thereby suppressing the proliferation of cardiac fibroblasts. Moreover, Astragaloside IV restrains the expression of TGF-β1 and Smad3, and upregulates Smad7 expression, indicating that ASG exerts antifibrotic effects by blocking the activation of the TGF-β/Smads signaling pathway (Wei et al., 2020). Saikosaponin A is a triterpene saponin isolated from Saikosaponin with anti-inflammatory, antioxidant, and anti-fibrotic effects (Fu et al., 2015). According to Liu et al. (2018b), Saikosaponin A alleviated the stress load-induced cardiac fibrosis. Interestingly, high-does Saikosaponin A could reduce the phosphorylation levels of smad2 and smad3 and the nuclear expressions of smad4, while low doses of Saikosaponin A blocked the TGF-β-induced EndMT, indicating that different doses of Saikosaponin A can attenuate the cardiac fibrosis process by regulating the TGF-β/smad signaling pathway and inhibiting the EndMT.

#### 4.1.5 Polyphenols

Resveratrol is a polyphenolic compound widely discovered in a variety of medicinal and edible plants, with anti-inflammatory, antioxidant, and anti-apoptotic pharmacological effects (Gambini et al., 2015). Zhu et al. (2022) found that TGF-β1, *a*-SMA, ColI, and Smad3 were significantly expressed in HG-induced H9c2 cells and were downregulated by resveratrol treatment. Such findings reveal that resveratrol reduces the ECM deposition by restraining the activation of TGF-β1/Smad3 signaling pathway, thereby alleviating the HG-induced cardiac fibrosis.

#### 4.1.6 Others

Emodin is a derivative of anthraquinones, with a range of biological activities. According to the report, emodin can relieve the TGF-β1-induced activation of cardiac fibroblasts and the ECM accumulation *in vitro*. Moreover, emodin can inhibit the activation of several classical (Smad2/3) and non-classical (ERK1/2) signaling pathways. This indicates that emodin is an effective anti-cardiac fibrosis therapeutic agent that can intervene the development of cardiac fibrosis, by regulating different signaling pathways (Carver et al., 2021). Higenamine is an active ingredient extracted from a variety of Chinese herbal medicines, such as aconite root, nandina bamboo, and scutellaria barbata. Higenamine can inhibit both TAC-induced and ISO-induced cardiac fibrosis in addition to blocking the cardiac fibroblasts conversing to myofibroblasts by suppressing the TGF-β1/Smad signaling pathway, thereby improving cardiac fibrosis and dysfunction (Zhu et al., 2021) (Table 4).

**TABLE 4:** Mechanism of Chinese herbal extracts or natural compounds interfering with cardiac fibrosis by regulating TGF-signaling pathway.

Classify	Natural products	Targets of research	Efficacy	References
Alkaloids	Neferine	collagen I and III 、 TGF-β1	Restrain the migration of CFs	Zhao et al. (2010)
		TGF-β1-Smad、ERK、p38MAPK	Improving myocardial function in diabetic mice	Liu et al. (2016)
	Picrasidine	TGF-β1、Smad2/3	Improving the cardiac compliance and cardiac function	Zhang et al. (2018b)
Terpenoids	Zerumbone	TGF-β1、p-Smad2/3、MMP-2/9、Smad7	Inhibits the conversion of cardiac fibroblasts to myofibroblasts	Li et al. (2022c)
	Ginkgolide B	ERK1/2、JNK、38MAPK	Restraining the development of diabetic MF	Wu et al. (2013)
				Jang et al. (2020)
	*Centella asiatica*	Smad2/3、Smad7	Alleviating the overburden pressure-induced cardiac fibrosis	Lu et al. (2018)
	Betulinic acid	Smad2/3、TGF-β1	Relieving the hyperglycaemia-induced cardiac fibrosis	Jiang et al. (2017)
Flavonoids	Daidzein	Smad2/3、TGF-β1	Suppressing the development of cardiac disease	Yu et al. (2020)
	Apigenin	miR-122-5p、TGF-β1、Smad2/3、HIF-1α、Smad7	Anti-fibrosis effect	Zhou et al. (2016)
				Feng et al. (2021)
	Glycyrrhizin	TGF-β1、Smad2	Relieved fibrosis in mice and cardiomyocytes by suppressing	Li et al. (2021b)
	Silymarin	TGF-β1、Smad2/3、Smad7	Decreasing MF and collagen deposition	Meng et al. (2019)
	Baicalin	TGF-β、Smad2/3、Smad4	Anti-fibrosis effect	Xiao et al. (2018)
Saponins	Astragaloside IV	TGF-β1、Smad3、Smad7	Anti-fibrosis effect	Wei et al. (2020)
	Saikosaponin A	smad2、smad3、smad4	Attenuate the cardiac fibrosis and inhibiting the EndMT process	Fu et al. (2015)
				Liu et al. (2018b)
Polyphenols	Resveratrol	TGF-β1、Smad2、Smad3	Reducing ECM deposition and alleviating HG-induced cardiac fibrosis	Gambini et al. (2015)
				Zhu et al. (2022)
Others	Emodin	SMAD2/3、Erk1/2	Anti-cardiac fibrosis	Carver et al. (2021)
	Higenamine	TGF-β1、Smad	Improving cardiac fibrosis and dysfunction	Zhu et al. (2021)

### 4.2 Anti-cardiac fibrosis herbal remedies

According to the previous studies, there are many herbal formulas for inhibiting the progression of cardiac fibrosis by regulating the TGF-β/Smad signaling pathway. As indicated by one research, Fu fang Zhen Zhu Tiao Zhi (FTZ) could greatly downregulate the expressions of *a*-SMA, Col1A2, Col-III, and CTGF, thereby inhibiting the ECM deposition and the cardiac fibrosis. Moreover, FTZ also attenuated the proliferation and migration of cardiac fibroblasts by restraining the activation of the TGFβ1-Smad2/3 signaling pathway and suppressing the collagen synthesis, indicating that FTZ possessed great therapeutic potential in the treatment of cardiac fibrosis (Zhang et al., 2022c). Similar to the researches above, the Heart-Protective Soup can inhibit the expressions of Col-Ⅰ and Col-Ⅲ in adriamycin-induced dilated cardiomyopathy, in addition to reducing the expressions of TGF-β1 and Smad3, thereby promoting cardiac function and reducing cardiac fibrosis (Sun et al., 2017). In another research, the Kangxian Formula (KXF) was found to inhibit the AngII-induced proliferation and migration of cardiac fibroblasts in addition to reducing the expression of TGFβRI, TGFβRII, Smad2, Smad3, and Smad4 in cardiac fibroblasts, suggesting that KXF exerted a protective effect against cardiac fibrosis by blocking the activation of the TGF-β signaling pathway (Chen et al., 2021a). According to Su et al. (2020), Si-Miao-Yong-An Decoction (SMYAD) could decrease the expressions of *a*-SMA, Col, and ColIII, thereby reducing the ECM deposition. Moreover, SMYAD could block TGF-β1/Smad7 expression by suppressing the expressions of TGF-β1, Smad2, and Smad3 and upregulating the Smad7 expression. Thereby, progression of cardiac fibrosis can be inhibited by blocking TGF-β1/Smad pathway activation. Besides, Tao Hong Si Wu Tang (THSWD), a traditional formula consisting of Tao Ren, Hong Hua, Shu Di, Bai Shao, Chuan Xiong, and Angelica sinensis, was found to inhibit cardiac fibroblasts proliferation and collagen expression by restraining the activation of TGFβR1/Smad signaling pathway in a dose-dependent manner in a post-MI mouse model. These data indicated that THSWD inhibited cardiac fibroblasts proliferation and collagen expression, alleviated cardiac fibrosis, and improved cardiac function in mice after MI, by blocking activation of the TGFβR1/Smad signaling pathway (Tan et al., 2021). Similarly, in a model of radiation cardiac injury constructed by local irradiation of the precordial region at 25 Gy, Huangqi Shengmai Yin (HSY) was discovered to inhibit the epithelial proliferation, cardiac fibroblasts proliferation, and collagen deposition in irradiated tissues by blocking the binding of TGFβ1 to its receptor. Besides, it can maintain the balance between collagen synthesis and degradation by reversing the effects of radiation on MMP14 and TIMP1 expression and the balance between collagen synthesis and degradation (Cella et al., 2015; Gu et al., 2019) (Table 5).

**TABLE 5:** Mechanism of Chinese herbal medicine regulating TGF-signaling pathway and intervening cardiac fibrosis.

Herbal remedies	Targets of research	Efficacy	References
FTZ	TGFβ1、Smad2/3	Inhibiting the ECM deposition and the cardiac fibrosis	Zhang et al. (2022c)
BXD	TGF-β1、Smad3	Cardiac function and reducing cardiac fibrosis	Chen et al. (2020b)
KXF	TGFβRI、TGFβRII、Smad2、Smad3、Smad4	Anti-fibrosis effect	Chen et al. (2021a)
SMYAD	TGF-β1、Smad2、Smad3、Smad7	Anti-fibrosis effect	Su et al. (2020)
THSWD	TGFβR1、Smad	Anti-fibrosis effect	Tan et al. (2021)
HSY	MMP14、TIMP1	Balance between collagen synthesis and degradation	Cella et al. (2015)
			Gu et al. (2019)

### 4.3 Proprietary Chinese medicines against cardiac fibrosis

**FIGURE 3:**
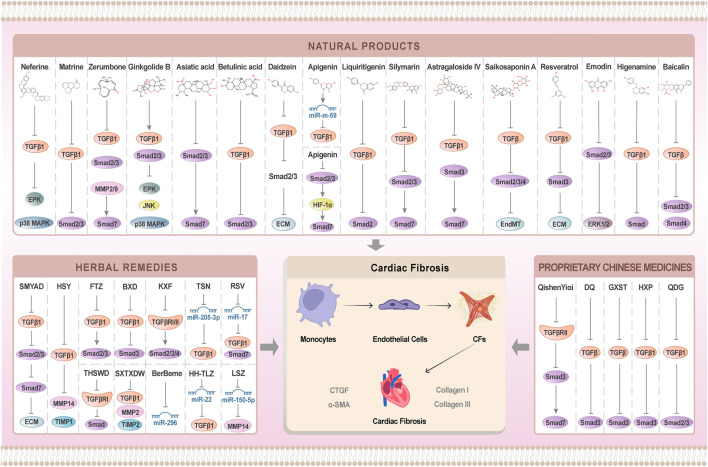
Mechanism of TCM intervention on cardiac fibrosis by regulating TGF-signaling pathway.

In recent years, under the guidance of the theoretical system of TCM, researchers have actively searched for the Chinese patent medicines that exert anti-cardiac fibrosis effects by regulating the TGF-β/Smad signaling pathway. For example, in a rat model of ascending aortic stenosis (AAS), Qi Shen Yi Qi Pill (QSYQP) was proven to be able to inhibit the expression of TGFβRII and Smad3, and up-regulate the expression of Smad7, thereby suppressing ECM deposition. Moreover, QSYQP also inhibited overburden pressure-induced cardiac fibrosis, with the mechanism associated with the inhibition of cardiomyocyte apoptosis and TGF-β1/Smad signaling pathway (Anwaier et al., 2022). It was shown that upregulation of the TGF-β1/Smad3 pathway may increase the susceptibility to differentiation of cardiac fibroblasts in MI and TAC rats. Treatment with Danqi Soft Capsule (DQ) and Guanxin Shutong Capsule (GXST) could decrease the expressions *a*-SMA, TGF-β1, and p-Smad3 greatly. It clarifies that DQ and GXST reversed the myofibroblast formation by regulating the TGF-β/Smad3 signaling pathway, thereby improving cardiac fibrosis in MI and TAC rats (Pan et al., 2021; Ma et al., 2022). Huo Xin Pill not only inhibited the ISO-induced proliferation, migration, and differentiation of cardiac fibroblasts, but also restrained the proliferation and migration of cardiac fibroblasts by suppressing TGF-β1/smads signaling pathway activation, thus exerting an anti-cardiac fibrosis effect (Peng et al., 2021). Furthermore, the Chinese patent medicine Qingxuan Jiangya decoction (QDG) was first used for the treatment of hypertension. According to a recent *in vivo* research, a medium dose of QDG (0.9 g/kg/day) greatly reduced the left ventricular ejection fraction, improved the cardiac function in SHR rats by reducing α, and restrained the differentiation and proliferation of cardiac fibroblasts by reducing the SMA expression and proliferating the cell nuclear antigen (PCNA). However, *in vitro* experiments presented that 6.25 and 12.5 μg/mL of QDG could inhibit the AngII-induced activation of the TGF-β1/Smad2/3 signaling pathway, thereby effectively reducing collagen deposition and inhibiting interstitial fibrosis (He et al., 2020; Chen et al., 2021b). HF is the swollen stage of various CVDs, and cardiac fibrosis can accelerate the deterioration of cardiac function in HF patients. As demonstrated by a recent *ex vivo* research, QSG inhibited the proliferation and differentiation of cardiac fibroblasts, and reduced the collagen deposition in the infarct margins of HF rats, by inhibiting the expression of TGF-β1 and Smad3 and up-regulating the expression of Smad7 (Guo et al., 2016; Zeng et al., 2019). Wu et al. (2021) observed 140 patients with IHF treated with Shexiang Tongxin Dropping Pills (STDP) )and found that the expressions of TGF-β1 and MMP2 were lower while the TIMP2 expression was higher when compared with the control group. The results revealed that STDP can not only regulate the dynamic balance of MMP2 and TIMP2 to maintain ECM homeostasis but also inhibit the TGF-β1 to impair the differentiation, migration, and proliferation of cardiac fibroblasts, which in turn hindered the cardiac fibrosis. Such results could be found in Table 6; Figure 3.

**TABLE 6:** Proprietary Chinese medicines regulating TGF-signaling pathway and intervening cardiac fibrosis.

Proprietary Chinese medicines	Targets of research	Efficacy	References
QSYQ	TGFβRII、Smad3、Smad7	Suppressing ECM deposition and anti-fibrosis effect	Anwaier et al. (2022)
DQ and GXST	TGF-β、Smad3	Improving cardiac fibrosis in MI and TAC rats	Ma et al. (2022)
			Pan et al. (2021)
HXP	TGF-β1、smads	Anti-fibrosis effect	Peng et al. (2021)
QDG	TGF-β1、Smad2/3	Educing collagen deposition, and inhibiting interstitial fibrosis	He et al. (2020)
			Chen et al. (2021b)
QSG	TGF-β1、Smad3、Smad7	Inhibited the proliferation and differentiation of CFs	Guo et al. (2016)
			Zeng et al. (2019)
SXTXDW	TGF-β1、MMP2、TIMP2	inhibit the progress of cardiac fibrosis	Wu et al. (2021)

### 4.4 Alternative therapies of TCM modulating ncRNAs and TGF-β/Smad signaling pathway against cardiac fibrosis

In the above sections, the research progress related to TCM and ncRNAs in cardiac fibrosis through modulation of TGF-β/Smad signaling pathways, respectively, is presented. These three aspects are not isolated but crosstalk each other, and TCM is also involved in the progression of cardiac fibrosis through modulation of ncRNAs and classical and non-classical TGF-β/Smad signaling pathways. A previous study reported that tanshinone IIA (TSN) protected the myocardium and inhibited cardiomyocyte hypertrophy. miR-205-3p and TGF-β1 were significantly downregulated after a two-week treatment with TSN. Transfecting miR-205-3p in cardiac fibroblasts could elevate the TGF-β1 expression and Col1a1 and Col3a1 were upregulated in TGF-β1-induced cardiac fibroblasts, which can be reversed by TSN treatment. It suggested that TSN enhances the cardiac function and attenuates the cardiac fibrosis after MI by upregulating miR-205-3p and thereby inhibiting TGF-β1 levels (Qiao et al., 2021). Furthermore, TGF- β1 is a key factor in progression of myocardial fibrosis, which promotes ECM deposition and thus plays an important role in cardiac remodeling. Zhang et al. found that resveratrol (RSV) inhibited the TGF-β1-induced cardiac fibroblasts proliferation and collagen secretion by a mechanism associated with silencing miR-17 or overexpression of Smad7, thus providing a new experimental basis for the efficacy of RSV in treating myocardial fibrosis (Zhang et al., 2018c). Another study showed that berberine can effectively inhibit left ventricle hypertrophy and cardiac fibrosis and improve the cardiac function (Liao et al., 2018). Zheng et al. (2020) investigated the effect of berberine on miR-29b expression in pressure-overloaded hypertrophic myocardium, and pre-applied it to treat the cardiac fibrosis. Finally, they confirmed that berberine (100 mg/kg/day) inhibited myocardial hypertrophy and fibrosis in stress overload models by up-regulating the expression of miR-29b and down-regulating the expression of its target genes. A study showed that Hong Hua (HH) improved cardiac function and ventricular remodeling and prevented the cardiac fibrosis in patients with ischemic cardiomyopathy., Ting Li Zi (TLZ) was proved to play an inhibitory effect on myocardial hypertrophy and fibrosis and corrected HF (Chen et al., 2019; Yu et al., 2019). Wang et al. (2020) observed the inhibitory effect of HH-TLZ on cardiac fibrosis in CHF mice after MI, and revealed that compared to the model group, CTL-TLZ (2.0 mg/mL) could upregulate the miRNA-22 and TGFβ-1 in myocardial tissue. In addition, the CTL-TLZ presented a role of downregulating COL1A1, COL3A1, and TGFβ-1 and upregulating miRNA-22 in cardiac fibroblasts, thus inhibiting the proliferation and collagen synthesis of cardiac fibroblasts. It suggests that the CTL-TLZ has an inhibitory effect on cardiac fibrosis in CHF mice after MI, and the mechanism may be related to the activation of miRNA-22/TGFβ-1 signaling pathway in fibroblasts.

Furthermore, TCM can regulate ncRNAs involved in the progression of cardiac fibrosis through TGF-β non-classical signaling pathways, such as the MMP signaling pathway. Gu et al. (2022) reported that Long Sheng Zhi Capsule (LSZ) inhibited the AngII-induced cardiac hypertrophy and fibrosis and rescued the expression of miR-150-5p treated with AngII; While miR 150-5p overexpression ameliorated the AngII-induced cardiac hypertrophy and fibrosis by attenuating the cardiac hypertrophy and fibrosis through MMP14 (Table 7; Figure 3).

**TABLE 7:** Mechanism of TCM against myocardial fibrosis by targeting TGF-β/Smad signaling pathway and ncRNAs.

Type	Traditional Chinese medicine	Targets of research	Efficacy	References
Natural products	TSN	miR-205-3p、TGF-β1	Alleviates cardiac fibrosis and improves ventricular remodeling following MI	Qiao et al. (2021)
	RSV	miR-17、Smad7	Inhibits CFs proliferation and collagen secretion	Zhang et al. (2018c)
	Berberine	miR-29b	Inhibits pressure overload-induced cardiac hypertrophy and myocardial fibrosis	Liao et al. (2018)
Traditional Chinese medicine prescription	HH-TLZ	COL1A1、COL3A1、TGFβ-1、miRNA-22	Inhibit the proliferation of CFs and collagen synthesis	Chen et al. (2019), Yu et al. (2019), Wang et al. (2020)
	LSZ	miR-150-5p、MMP14	Reduce cardiac hypertrophy and fibrosis	Gu et al. (2022)

## 5 Discussion

Cardiac fibrosis is characterized by excessive proliferation of cardiac interstitial fibroblasts, excessive collagen deposition, and abnormal distribution, which can lead to structural changes and systolic dysfunction in the heart, and further cause the development of advanced CVDs such as ischaemic heart disease, hypertension, and HF. Therefore, the development of cardiac fibrosis can be restrained partially or completely during its early stages of onset and progression by inhibiting or eliminating causative factors and using anti-fibrotic drugs.

Along with the research into the pathogenesis of cardiac fibrotic disease progresses, there are increasing evidences that the crosstalk between TGF-β and Smad signaling pathways plays an important role in progression of cardiac fibrosis. TGF-β1 is the key pro-cardiac fibrotic factor, Smad2 and Smad3 are central to interstitial fibrosis in the heart, and Smad7 is an important factor in anti-cardiac fibrosis. In view of the important role of TGF-β/Smad pathway in cardiac fibrosis, it is an attractive therapeutic approach to target TGF-β1 and its receptor and its downstream Smad proteins. Furthermore, as previously mentioned, ncRNAs can act in cardiac fibrosis by regulating the TGF-β-induced Smad classical signaling pathway and other non-classical signaling pathways. Besides, it was found in this work that ncRNAs did not regulate the development of cardiac fibrosis through a single signaling pathway, but rather acted as competitive RNAs *via* the process that their miRNA response elements worked as miRNA sponges and regulated their expression. Thus, lncRNAs, miRNAs, circRNAs, and miRNAs together regulated the TGF-β/Smad signaling pathway, thereby involving in progression of cardiac fibrosis. Based on the summary above, there is evidence that ncRNAs could be used as novel therapies for cardiac fibrosis. However, considering the different expressions and roles of ncRNAs in different samples, it is difficult to relate ncRNAs to the actual situation of each cardiac fibrosis patient. In the future, basic researches need to be translated into clinical trials, and ncRNAs can be used in regulating the TGF-β/Smad signaling pathway, for the real clinical treatment of cardiac fibrosis patients. Furthermore, there are numerous researches focused on the role of miRNAs and lncRNAs in regulating the TGF-β/Smad signaling pathway in cardiac fibrosis. Meanwhile, there are relatively few researches on the roles of circRNAs in regulating the TGF-β/Smad signaling pathway in cardiac fibrosis, indicating that more researches are needed in the future to explore the role of circRNAs in the regulation above.
